# Reduced Graphene Oxide-Wrapped FeS_2_ Composite as Anode for High-Performance Sodium-Ion Batteries

**DOI:** 10.1007/s40820-017-0183-z

**Published:** 2017-12-27

**Authors:** Qinghong Wang, Can Guo, Yuxuan Zhu, Jiapeng He, Hongqiang Wang

**Affiliations:** 1 School of Chemistry and Chemical Engineering, Jiangsu Key Laboratory of Green Synthetic Chemistry for Functional Materials, Jiangsu Normal University, Xuzhou, Jiangsu 221116 People’s Republic of China; 2 College of Chemistry and Environmental Science, Hebei University, Baoding, Hebei 071002 People’s Republic of China

**Keywords:** FeS_2_, Reduced graphene oxide (rGO), Enwrapping structure, Anode material, Sodium-ion battery

## Abstract

Iron disulfide is considered to be a potential anode material for sodium-ion batteries due to its high theoretical capacity. However, its applications are seriously limited by the weak conductivity and large volume change, which results in low reversible capacity and poor cycling stability. Herein, reduced graphene oxide-wrapped FeS_2_ (FeS_2_/rGO) composite was fabricated to achieve excellent electrochemical performance via a facile two-step method. The introduction of rGO effectively improved the conductivity, BET surface area, and structural stability of the FeS_2_ active material, thus endowing it with high specific capacity, good rate capability, as well as excellent cycling stability. Electrochemical measurements show that the FeS_2_/rGO composite had a high initial discharge capacity of 1263.2 mAh g^−1^ at 100 mA g^−1^ and a high discharge capacity of 344 mAh g^−1^ at 10 A g^−1^, demonstrating superior rate performance. After 100 cycles at 100 mA g^−1^, the discharge capacity remained at 609.5 mAh g^−1^, indicating the excellent cycling stability of the FeS_2_/rGO electrode. 
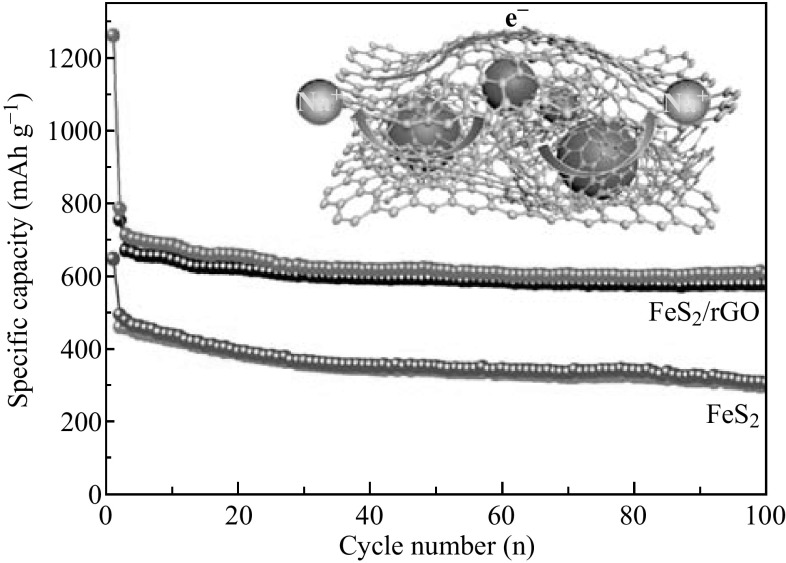

## Highlights

- Reduced graphene oxide-wrapped FeS_2_ (FeS_2_/rGO) composite was synthesized by a facile two-step method.
- The integral reduced graphene oxide networks not only connect the FeS_2_ nanoparticles but also prevent them from aggregating.
- As anodes for sodium-ion batteries, the FeS_2_/rGO composite delivers high specific capacity and good cycling stability.

## Introduction

Sodium-ion batteries (SIBs) have been considered to be promising candidates for large-scale energy storage systems, electric vehicles, and other portable devices because of their outstanding electrochemical performance and inexpensive characterization. As the most important parts of SIBs, electrolytes and electrodes have been extensively investigated. It has been demonstrated that polymer electrolytes (such as gel polymer electrolytes and ceramic electrolytes) may be desirable alternatives for liquid electrolytes because of their modularity and reliability in electrochemical devices [1, 2]. Various cathodes, including oxides (such as tunnel structure oxides and layered transition metal oxides), transition metal fluorides (typically FeF_3_), polyanionic compounds (such as NaFePO_4_, NaVPO_4_F, Na_3_M_2_(PO_4_)_3_ NASICON compounds), Prussian blue analogues, and organic electrodes, have displayed outstanding sodium storage performance [3]. Meanwhile, significant progress has been achieved in the area of anodes for SIBs. It has been revealed that carbon materials, metals, alloys, and metal oxides/sulfides are promising anode materials for SIBs [4–6]. Among these materials, iron sulfides, such as FeS_2_ [7–9], FeS [10] and Fe_1−x_S [11, 12], have attracted much attention due to their advantages of abundant reserves, non-toxicity, low cost, and high theoretical capacity (894 mAh g^−1^ for FeS_2_, based on the conversion reaction of FeS_2_ + 4Na ↔ 2Na_2_S + Fe). However, the intrinsically low conductivity and notable volume change during the charge–discharge process greatly restrain its rate performance and cycling stability, restricting its further commercialization.

To solve these problems, Chen’s group tuned an electrolyte and applied a higher voltage cutoff to improve the electrochemical performance of Na/FeS_2_ and Li/FeS_2_ cells [13, 14]. Numerous studies have revealed that constructing nanostructured materials can greatly reduce the electron/ion transport pathways and effectively buffer the large volume expansion during electrochemical processes, thus improving the reversibility and rate capability of FeS_2_ anode materials [15, 16]. Moreover, the combination of a carbon or a polypyrrole (ppy) modification strategy to form a coating or an embedded structure (such as FeS_2_/C [17–20], ppy@MoO_3_ [21–23], ppy@V_2_O_5_ [24]) would help prevent aggregation and improve the conductivity of the electrode materials, thus enhancing the cycling and rate performance. For example, Liu et al. designed FeS_2_@C nanoboxes and obtained discharge capacity of 511 mAh g^−1^ at 100 mA g^−1^ after 100 cycles [25]. Graphene is a highly conductive ultrathin nanosheet, with a large surface area and high flexibility, which is commonly used as modification material. In previous studies, FeS/reduced graphene oxide (rGO) [26], FeS_2_/rGO [27], Fe_3_O_4_/rGO [28], Fe_2_O_3_/rGO [29], and LiFePO_4_/rGO [30, 31] composites have been fabricated and used in lithium-ion batteries (LIBs). It is demonstrated that enwrapping anode materials in a conductive network is a favorable strategy to enhance the rate capability. However, the FeS_2_/graphene composite with an enwrapping structure for SIBs has not been reported.

Here, we report a two-step method for the preparation of a novel rGO-wrapped FeS_2_ (FeS_2_/rGO) composite for SIBs. Structural and morphological characterization revealed that the FeS_2_ nanoparticles are evenly surrounded in the interconnected rGO networks. The composite displayed superior sodium storage performance even at high charge–discharge current densities.

## Experimental

### Materials Synthesis

FeS_2_/rGO was synthesized via a hydrothermal method, followed by a sulfurization process. All chemicals were of analytical grade and used without further purification.

*Synthesis of Fe*_*3*_ *O*_*4*_ */rGO composite* In a typical synthesis, 0.04 g of graphene oxide was dispersed in 65 mL of deionized water by sonication. Then, 0.4 g of Fe(NO_3_)_3_·9H_2_O was dissolved in the above suspension and stirred for 4 h at 70 °C. Following this, 5 mL of N_2_H_4_·H_2_O was added to the above system, and the solution was sealed in a 100-mL Teflon-lined stainless-steel autoclave for hydrothermal reaction at 150 °C for 6 h. Finally, the rGO-wrapped Fe_3_O_4_ composite was collected by centrifugation, washed with water and ethanol three times, and dried at 70 °C in a vacuum for 12 h.

*Synthesis of FeS*_*2*_ */rGO Composite* The as-prepared Fe_3_O_4_/rGO and sulfur powder in a weight ratio of 1:2 were mixed and pressed into a small tablet and sealed in a small quartz tube under Ar atmosphere. Then, the quartz tube was heated at 150 °C for 2 h and subsequently at 550 °C for 6 h in a quartz tube reactor. After cooling down and washing with CS_2_ to remove the residual sulfur powder, the final FeS_2_/rGO composite was obtained. For comparison, FeS_2_ nanoparticles were prepared using the same method without the addition of rGO.

### Materials Characterization

The crystal structures of the as-prepared samples were characterized by powder X-ray diffraction (XRD) using Cu Ka radiation. The morphologies were investigated using field-emission scanning electron microscopy (SEM) on a JEOL JSM-7500FA system and transmission electron microscopy (TEM) on a Philips Tecnai 20 (200 kV). TG thermal nitrogen adsorption–desorption isotherms of the samples were obtained on a Quantachrome Autosorb-IQ2 analyzer at 77 K. Specific surface areas were measured by Brunauer–Emmett–Teller (BET) analysis.

### Electrochemical Measurements

Electrochemical measurements were conducted using CR2032 two-electrode coin cells, with sodium metal as the counter and reference electrodes and glass fiber as the separator. The working electrodes were made by pasting a slurry on copper foil, followed by drying in vacuum at 80 °C for 12 h. The slurry was prepared by mixing active materials, Super P, and carboxymethyl cellulose in the weight ratio of 8:1:1. A solution of 1 M NaClO_4_ in ethylene carbonate/propylene carbonate (v/v = 1/1) with 5 wt% fluoroethylene carbonate additive was used as the electrolyte. Cyclic voltammetry (CV, 0–2.5 V, 0.1 mV s^−1^) tests and electrochemical impendence spectroscopy (EIS, with 5 mV amplitude in a frequency range from 100 kHz to 0.01 Hz at open-circuit potential) tests were conducted on a Biologic VMP-3 electrochemical workstation. The galvanostatic charge–discharge curves, cycling performance, and rate capabilities of the electrode materials were tested on a LAND Battery Test System, in the voltage range of 0.01–2.3 V. All the tests were carried out at room temperature.

## Results and Discussion

**Fig. 1:**
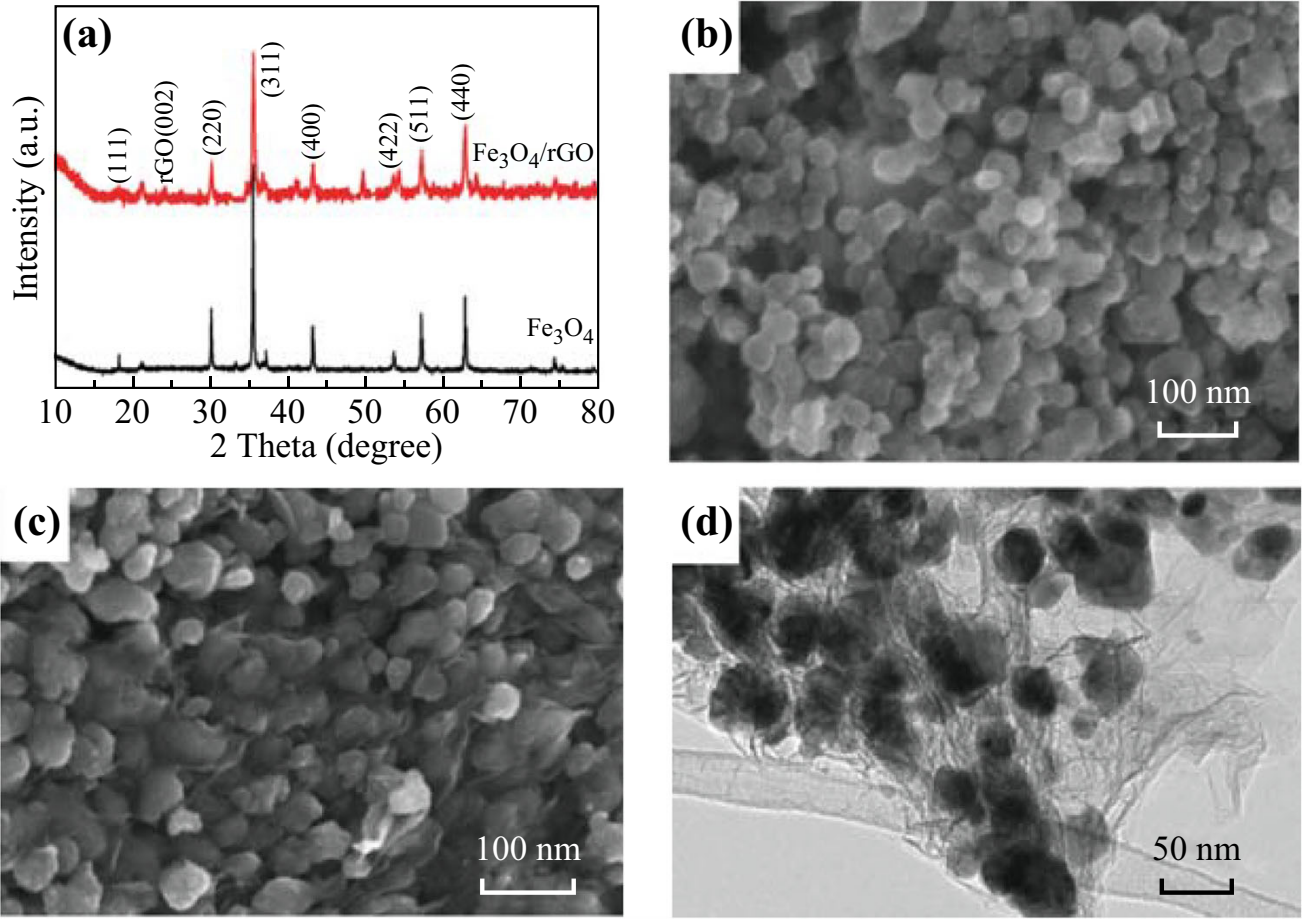
**a** XRD patterns of the as-prepared Fe_3_O_4_ and Fe_3_O_4_/rGO precursors, **b** SEM image of Fe_3_O_4_, **c** SEM image, and **d** TEM image of Fe_3_O_4_/rGO

The crystal structures and morphologies of the as-prepared Fe_3_O_4_/rGO and Fe_3_O_4_ precursors are characterized by XRD, SEM, and TEM. From Fig. 1a, it can be seen that the main peaks in the XRD patterns are indexed to magnetite Fe_3_O_4_ (JCPDS card No. 75-0449). For the Fe_3_O_4_/rGO composite, a weak peak at about 22.5° is detected, which can be indexed to rGO. From Fig. 1b, c, one can see that both the precursors are mainly composed of uniform nanoparticles about 80 nm in diameter. Figure 1c shows that each nanoparticle is surrounded by thin graphene nanosheets. The TEM image shown in Fig. 1d further confirms the enwrapped structure of the composite. Moreover, it is obvious that each nanoparticle is connected by rGO to form an integral 3D network.

**Fig. 2:**
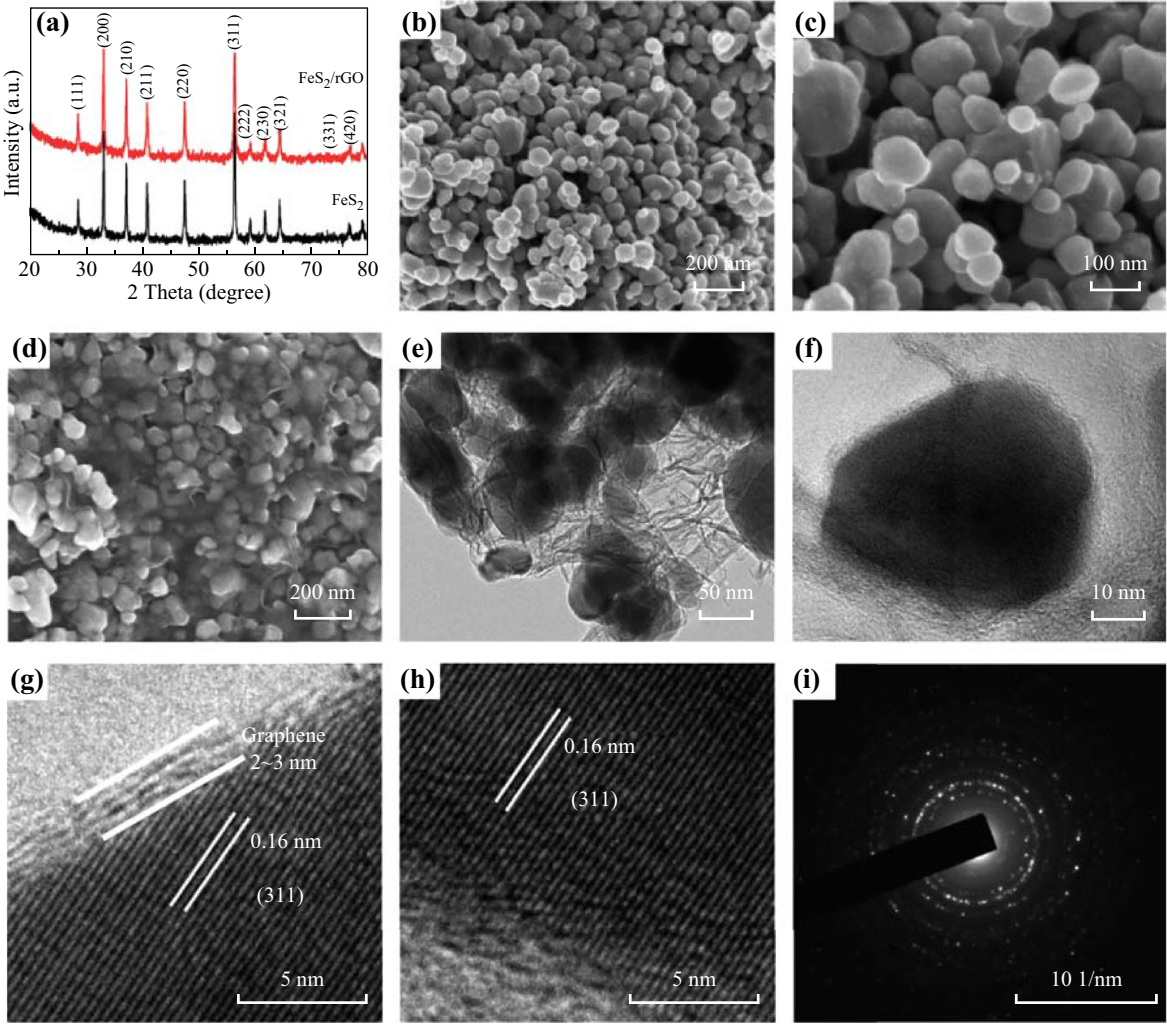
**a** XRD patterns of the as-prepared FeS_2_ and FeS_2_/rGO composite, **b**, **c** SEM images of FeS_2_, **d** SEM image, **e**, **f** TEM images, **g**, **h** HRTEM images, and **i** SAED pattern of FeS_2_/rGO composite

Figure 2 shows the crystal structure and morphologies of the as-prepared FeS_2_ and FeS_2_/rGO samples. As shown in Fig. 2a, both the samples show high-intensity XRD peaks, all of which can be indexed to pyrite FeS_2_ (JCPDS card No. 06-0710), demonstrating the high purity and good crystallinity of the two samples. Figure 2b, c shows that the FeS_2_ sample is composed of irregular particles like the Fe_3_O_4_ precursor. However, it can be observed that the particles tend to aggregate and become larger than the precursor, which is caused by the sulfuration process. Figure 2d shows that the general morphology of the FeS_2_/rGO composite is similar to that of the Fe_3_O_4_/rGO precursor. The TEM image shown in Fig. 2e further reveals that the FeS_2_ nanoparticles continue to be evenly dispersed in the graphene networks and that the particle size remains largely unchanged, compared to its precursor. Figure 2f shows that each nanoparticle is surrounded by graphene, which effectively prevents the aggregation of the FeS_2_ nanoparticles. The thickness of the graphene layer is 2–3 nm (Fig. 2g). High-resolution TEM images (HRTEM, Fig. 2g, h) display clear lattice fringes with an interplane distance of 0.16 nm, corresponding to the (311) plane of pyrite FeS_2_. The selected-area electron diffraction (SAED) pattern of FeS_2_/rGO (Fig. 2i) shows well-defined rings, indicating that the as-prepared FeS_2_ is polycrystalline.

**Fig. 3:**
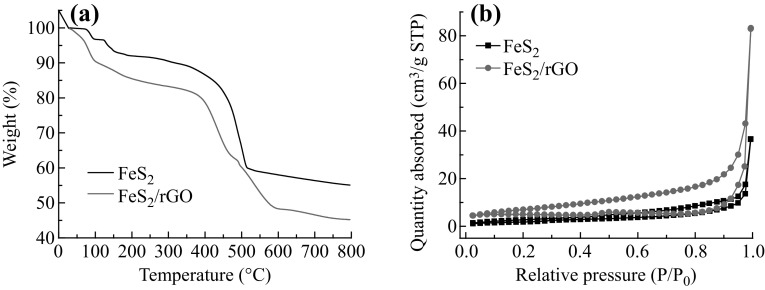
**a** TG curves and **b** N_2_ adsorption–desorption curves of FeS_2_ and FeS_2_/rGO composite

According to the N_2_ adsorption–desorption measurements (Fig. 3b), the specific surface areas of FeS_2_ and FeS_2_/rGO are 25.6 and 58.1 m^2^ g^−1^, respectively, indicating that the introduction of rGO significantly increases the surface areas. To determine the rGO content in the composite, thermogravimetric analysis is carried out in an air atmosphere (Fig. 3a). Both the samples display a minor weight loss (∼ 6–8%) under 200 °C, which is due to the vapor of the residual water in the materials. Then a large weight loss of about 35% is observed in the range 400–500 °C for pure FeS_2_, which corresponds to the conversion of FeS_2_ to Fe_2_O_3_. (The theoretical weight loss is ∼ 33.3%.) For FeS_2_/rGO, a more significant weight loss of about 42% is observed between 400 and 600 °C, which may be caused by the phase change of FeS_2_ to Fe_2_O_3_ and rGO to carbon dioxide. Based on the thermogravimetric analysis, the weight content of FeS_2_ in the FeS_2_/rGO composite can be calculated to be about 79.1%. According to the above analysis, the FeS_2_/rGO composite contains integral nanostructures, with the FeS_2_ nanoparticles enwrapped in the 3D rGO networks. This unique structure endows the composite with high structural stability and super electron conductivity, which may be beneficial for the cycling stability and rate performance of the FeS_2_ electrode material for sodium storage.

**Fig. 4:**
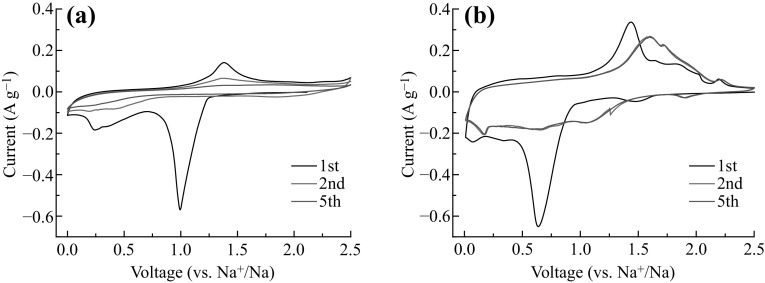
CV curves of **a** FeS_2_ and **b** FeS_2_/rGO

Figure 4a, b shows the cyclic voltammetry (CV) curves of pure FeS_2_ and the FeS_2_/rGO composite at a scan rate of 0.1 mV s^−1^ between 0 and 2.5 V (vs. Na/Na^+^). During the initial cathodic scan, a large peak appears at 1.0 V and a broad peak appears at 0.25 V for the FeS_2_ electrode, which corresponds to Na^+^ intercalation and the formation of the Na_x_FeS_2_ (*x* < 2) phase, Fe and Na_2_S, and the formation of a solid-electrolyte interface (SEI) layer [13, 16, 32]. For the FeS_2_/rGO electrode, a large peak at ~ 0.65 V and a small peak at ~ 0.1 V are detected, which may be due to a similar electrochemical process with the FeS_2_ electrode. The differences in the peaks of the two samples may be caused by the nanostructure and the introduction of rGO. During the subsequent anodic scan, the peaks observed at ~ 1.4 and ~ 1.8 V can be attributed to be the desodiation process, with the formation of Na_2_FeS_2_ and Na_2−x_FeS_2_ [19]. During the subsequent cycles, the CV curves are quite different from those in the initial cycle, which may be due to the irreversible formation of the SEI layer and the decomposition of the electrolyte [19, 33–35]. It can be observed that the FeS_2_/rGO electrode shows much better repeatability and a larger closed curve area than those of the pure FeS_2_ electrode, demonstrating its much better cycling stability and higher specific capacities.

**Fig. 5:**
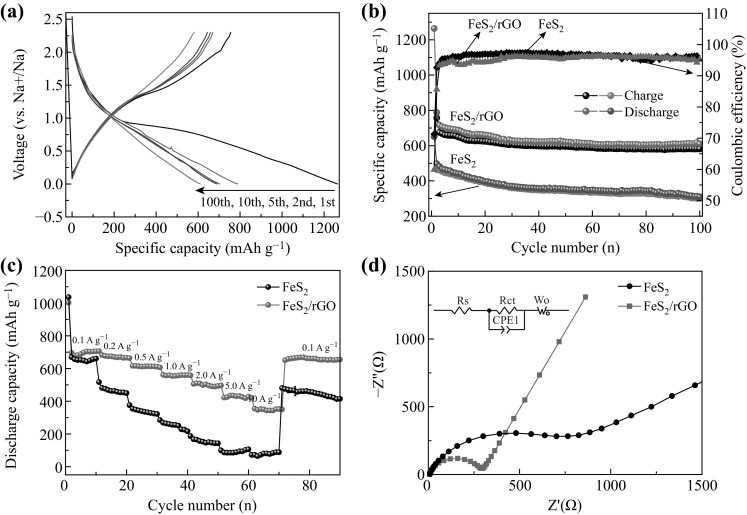
**a** Galvanostatic charge–discharge curves of FeS_2_/rGO, **b** cycle life and coulombic efficiencies, **c** rate performance, and **d** Nyquist plots for FeS_2_ and FeS_2_/rGO composite

Figure 5a presents the charge–discharge curves of FeS_2_/rGO electrode at a current density of 100 mA g^−1^. An initial discharge plateau at ~ 1.0 V (vs. Na/Na^+^) and charge plateau at ~ 1.3 V are observed, which are in good agreement with the CV curves. In the subsequent cycles, the charge–discharge curves do not change much, showing good electrochemical reversibility. The cycling performances of the two samples are further evaluated at 100 mA g^−1^. As shown in Fig. 5b, both the electrodes have quite good cycling stability. However, the FeS_2_/rGO electrode has obviously higher specific capacities than does the pure FeS_2_ electrode, which may be due to the higher utilization of the active materials after the introduction of rGO. The FeS_2_/rGO composite displays a high initial discharge capacity of 1263.2 mAh g^−1^ and charge capacity of 759.4 mAh g^−1^, showing a low coulombic efficiency of 60.1%, which is mainly caused by the irreversible formation of the SEI layer and electrolyte decomposition in the initial cycle. Moreover, the dissolution of sodium polysulfides into organic liquid electrolytes causes a parasitic redox shuttle, leading to unfavorable side reactions with sodium, reducing the charging efficiency and resulting in serious capacity decay [36–38]. In the following cycles, the coulombic efficiency increases over 95%. From the second cycle, the discharge and charge capacities are stable and remain at 609.5 and 581.7 mAh g^−1^, respectively, after 100 cycles.

The rate capability of the two FeS_2_ electrodes is evaluated using varying current densities from 0.1 to 10 A g^−1^ and back to 0.1 A g^−1^. As shown in Fig. 5c, the average specific capacities for FeS_2_/rGO electrodes are 705, 672, 613, 555, 496, 426, and 344 mAh g^−1^ at 0.1, 0.2, 0.5, 1, 2, 5, and 10 A g^−1^, respectively, which are remarkably higher than those for pure FeS_2_ electrode, demonstrating its superior rate performance. When the current density is altered back to 0.1 A g^−1^, the reversible capacity remains at ~ 655 mAh g^−1^ after 90 cycles, further confirming the excellent cycling stability of the FeS_2_/rGO composite. We further investigate the electrode process kinetics of the two materials through EIS. As shown in Fig. 5d, both the Nyquist spectra are composed of a semicircle in the high-frequency region and an inclined line in the low-frequency region. The bigger semicircle for the FeS_2_ electrode illustrates the poor electrical conductivity of the active materials. According to the Z-view program in the Sai software set, *R*_ct_ for FeS_2_ and FeS_2_/rGO electrodes is 1055.1 and 291.9 Ω, respectively, illustrating the better charge transfer kinetics of the FeS_2_/rGO electrode.

**Fig. 6:**
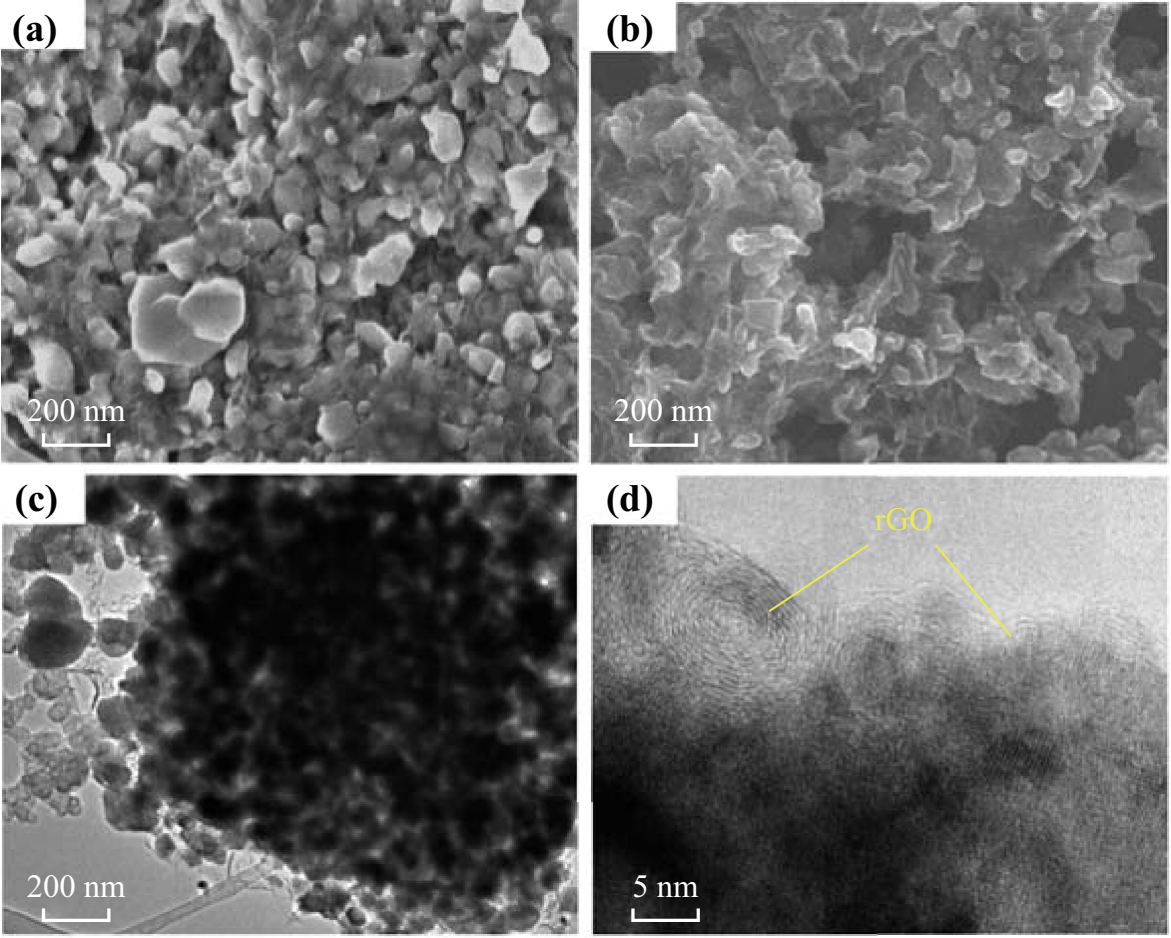
**a** SEM image of freshly prepared FeS_2_/rGO electrode, **b** SEM image, and **c**, **d** TEM images of FeS_2_/rGO electrode after 100 charge–discharge cycles

The FeS_2_/rGO composite displays much higher specific capacity and better rate capability than does the pure FeS_2_ electrode. It is inferred that several features may contribute to the excellent electrochemical properties. First, the intimate contact of the FeS_2_ nanoparticles with rGO and the integral conductive rGO networks provide a facile electron transport pathway, ensuring good rate performance [27, 30]. Second, the unique enwrapping structure can effectively improve the structural stability and buffer the volume change of FeS_2_ during the charge–discharge process [26, 28]. To investigate the structural stability, the nanostructures of the freshly prepared FeS_2_/rGO electrode and the FeS_2_/rGO electrode after 100 cycles are investigated by SEM and TEM. From Fig. 6a, it can be seen that the morphology of the FeS_2_/rGO composite does not change. After 100 sodiation–desodiation cycles, the nanoparticles are not very regular but are still enwrapped in the graphene networks (Fig. 6b, c). The high-resolution TEM test shows that the nanoparticles transform into smaller nanocrystals (Fig. 6d), which are still surrounded by rGO. It is obvious that the graphene network can effectively prevent the collapse of the structure and the aggregation of FeS_2_ nanoparticles, thus improving the cycling stability of the FeS_2_/rGO composite. Moreover, the improvement of the BET surface area increases the contact area between the active material and the electrolyte, which helps improve the utilization of active materials, endowing the FeS_2_/rGO composite with high specific capacitance.

## Conclusions

In summary, an rGO-wrapped FeS_2_ composite has been successfully synthesized via a hydrothermal method, followed by sulfuration, and used as an anode for SIBs. The well-dispersed rGO constructs 3D conductive networks and markedly increases the BET surface area and conductivity of the FeS_2_ nanoparticles. Thus, the FeS_2_/rGO composite displays an initial discharge capacity of 1263.2 mAh g^−1^ at 100 mA g^−1^ and a high discharge capacity of 344 mAh g^−1^ at 10 A g^−1^. Moreover, the enwrapping structure helps in preventing the aggregation of the FeS_2_ nanoparticles during the electrochemical process, contributing to the excellent cycling stability. After 100 cycles, the discharge capacity is 609.5 mAh g^−1^. We believe that our strategy could be extended to the fabrication of other high-performance metal sulfide/rGO composites for LIBs or SIBs.
